# Real-world data analysis of immune checkpoint inhibitors in stage III-IV adenocarcinoma and squamous cell carcinoma

**DOI:** 10.1186/s12885-022-09843-3

**Published:** 2022-07-13

**Authors:** Meiling Sun, Huaijun Ji, Ning Xu, Peng Jiang, Tao Qu, Yu Li

**Affiliations:** 1 Department of Pulmonary and Critical Care Medicine, Qilu Hospital, Cheeloo College of Medicine, Shandong University, No. 107 Wenhua Xilu, Jinan, 250012 Shandong China; 2 Department of Respiratory Medicine, Weihai Municipal Hospital, 70 Heping Road, Weihai, 264200 Shandong China; 3 Department of Thoracic Surgery, Weihai Municipal Hospital, 70 Heping Road, Weihai, 264200 Shandong China

**Keywords:** Immune checkpoint inhibitors (ICIs), PD-L1 inhibitor, NSCLC, Objective response rate, Disease control rate, Median progression-free survival

## Abstract

**Background:**

This study was designed to investigate the clinical application, efficacy, and safety of immune checkpoint inhibitors (ICIs) in the treatment of lung cancer in the real world.

**Methods:**

A retrospective, observational analysis was conducted on patients treated with ICIs in four tertiary hospitals in the region from January 2015 to March 2021, to evaluate the clinical efficacy of ICIs single-agent or combined chemotherapy and anti-vascular drugs in the first-line or second-line treatment of patients with lung cancer.

**Results:**

Three hundred and fifteen patients were enrolled in this study. In patients with stage III-IV adenocarcinoma and Squamous cell carcinoma, the objective response rate (ORR) and disease control rate (DCR) were 35.5% (87/245) and 93.5% (229/245), respectively, the median progression-free survival (PFS) was 10.8 months, and the median overall survival (OS) was not reached. A total of 132 patients received ICIs as the first-line treatment, the median treatment cycle was 8 cycles (2–20 cycles), the short-term efficacy ORR was 38.6%, DCR was 93.9%, and the median PFS was 11.4 months. One hundred thirteen patients received ICIs treatment as second-line treatment, the median treatment cycle was five cycles (2–10 cycles), the short-term efficacy ORR was 31.9%, DCR was 92.9%, and the median PFS was 10.0 months. There were no statistically significant differences in ORR, DCR, or median PFS with ICIs as the first-line treatment compared with the second-line treatment(*P* > 0.05). The results of subgroup analysis showed that Eastern Cooperative Oncology Group performance status (ECOG PS), epidermal growth factor receptor (EGFR) mutation status, pathological type and number of treatment lines were not correlated with median PFS(*P* > 0.05). However, there were statistically significant differences in programmed death-ligand 1(PD-L1) expression, corticosteroid interference, and antibiotic (Abx) treatment among all groups (*P* < 0.05). In terms of safety, the overall incidence of adverse reactions in 315 patients was 62.5%, and the incidence of immune-related adverse events (irAEs) was 13.7%. Grade 1–2 and 3–4 incidence of adverse events were 34.9 and 27.65%, respectively. There were four patients who experienced fatal irAEs, two cases were liver damage leading to liver failure, one case was immune related pneumonia, and one case was immune related myocarditis.

**Conclusion:**

In the real world, ICIs has a good effect on patients with lung cancer and significantly improves ORR and PFS.

## Background

Lung cancer remains the malignant tumor with the highest morbidity and mortality in China and the world, among which non-small cell lung cancer (NSCLC) accounts for approximately 85% of cases [1]. For the traditional treatment model, the 5-year survival rate of advanced lung cancer is only approximately 5%, and after first-line antitumor treatment, the disease is still not under control, and metastasis occurs in some patients [2]. Immunotherapy, represented by ICIs, has changed the treatment mode of a variety of advanced malignant tumors, especially in the field of lung cancer treatment, which has played an important role in the long-term survival effect of some patients with advanced lung cancer and greatly improved the prognosis of patients with lung cancer.

Based on the results of KEYNOTE-024, the Food and Drug Administration (FDA) approved pembrolizumab combined with chemotherapy for the first-line treatment of metastatic NSCLC with PD-L1(TPS ≥ 50%) [3]. Randomized clinical trial results from CheckMate 057 [4], KEYNOTE-001 [5] and KEYNOTE-010 [6] showed that nivolumab, atezolizumab, and pembrolizumab were superior to chemotherapy in ORR, PFS, and OS. For nondriver gene mutation/fusion advanced NSCLC with PD-L1 expression < 50%, the results of KEYNOTE-189 [7], IMpower-150 [8], and other trials have shown that ICIs combined with chemotherapy could bring more survival benefits to patients than chemotherapy.

This study collected the case data of 315 patients with lung cancer who received ICIs in four hospitals in Shandong from January 2015 to March 2021, aimed to explore the clinical efficacy and safety of ICIs in first-line or second-line treatment of lung cancer patients in the real world and possible factors related to efficacy and side effects.

## Methods

### Data collection

We retrospectively collected patients with lung cancer who received ICIs in a number of hospitals in Shandong from January 2015 to March 2021. The patients’ age, sex, past history, smoking history, pathological type, ECOG PS, TNM staging, tumor metastasis (brain/bone/liver metastasis), driver gene detection, PD-L1 expression, corticosteroid or Abx interference were collected. Treatment included ICIs drugs and methods, number of lines (1 line/2 lines), clinical efficacy, and adverse reactions. All data were obtained from follow-up and clinical medical records.

### Patient selection

(1) Inclusion criteria: an ECOG PS of 0 to 2, survival time > 3 months; pathological or cytological diagnosis of lung cancer; and at least two cycles of ICIs. (2) Exclusion criteria: no clear pathological diagnosis information; patients with other tumors at the same time; ECOG PS > 2; no efficacy evaluation after ICIs treatment; unstable brain metastases; autoimmune diseases. (3) The inference of corticosteroids was defined as receiving no less than 10 mg of prednisone equivalent for more than 20 days from the first day of ICIs treatment. (4) Evaluation of Abx treatment status: Patients who received Abx within 1 month before or after the first administration of ICI were defined as the Abx treatment group.

### Efficacy assessment

This study was a descriptive analysis of the efficacy of first-line and second-line immunotherapy in stage III-IV adenocarcinoma and Squamous cell carcinoma. According to the Response Evaluation Criteria in Solid Tumors (RECIST) version 1.1; efficacy, prognostic evaluation, and adverse reaction grading were evaluated. The evaluation was based on imaging examinations, and efficacy evaluation was performed at least once every two courses. An overall response was defined as a complete response (CR), partial response (PR), stable disease (SD), or progressive disease (PD). ORR = (CR + PR)/ (CR + PR + SD + PD) × 100%; DCR = (CR + PR + SD)/(CR + PR + SD + PD) × 100%; PFS was defined as the time from the start of ICIs initiation to the first event (tumor progression or death from any cause). OS was defined as the time from the start of iICIs to death from any cause. At each patient review, safety assessment was carried out through laboratory-related tests. Blood tests included routine blood tests, biochemistry, and corticosteroid measurements. IrAEs are judged according to version 4.03 of the National Cancer Institute Common Terminology Criteria for Adverse Events (NCI-CTCAE) and are divided into grades I-V. In this study, grades III-IV were defined as moderate to severe adverse reactions, and grade V was defined as death.

### Follow-up

The patients were followed up by consulting outpatient records, hospitalization medical records, and telephone inquiries. The last follow-up date was March 31, 2021. The content of the follow-up included the general condition of the patient, tumor treatment, disease progression, and treatment side effects. Those who were lost to follow-up and those who did not die were treated according to the cutoff value, and the cutoff time was the last follow-up time at which they were confirmed not dead. If the follow-up was lost, the time of the last follow-up was recorded.

### Statistical analysis

All statistical analyses were performed with SPSS ver. 23.0. Data were summarized using descriptive statistics or contingency tables for demographic and baseline characteristics, response measurements, and safety measurements. ECOG PS, EGFR mutations, PD-L1 expression levels, pathological type, corticosteroid interference, Abx treatment and lines of therapy were included in the univariate analysis. All survival analyses were estimated using Kaplan–Meier curves and compared using the log-rank test. *P* < 0.05 was considered statistically significant.

### Ethical statement

This program was performed in accordance with the principles of good clinical practice and was approved by the institutional review board of each hospital. All patients provided written informed consent before participation.

## Results

### Baseline characteristics

From January 2015 to March 2021, a total of 400 patients were screened, and 315 lung cancer patients who met the criteria were included. The baseline demographic and clinical characteristics are shown in Table 1.

**Table 1:** Baseline characteristics of all patients

Characteristic	N	Percentage
**Sex**		
Male	254	80.6
Female	61	19.4
**Comorbidities**		
Hypertension	105	33.3
Diabetes mellitus	46	14.6
Coronary heart disease	37	11.7
**Smoking history**		
Yes	216	68.6
No	99	31.4
**ECOG PS**		
0–1	285	90.5
2	30	9.5
**Histology**		
Adenocarcinoma	135	42.9
Squamous cell carcinoma	117	37.1
Small cell carcinoma	45	14.3
Large cell carcinoma	6	1.9
Unknown	12	3.8
**Clinical stage**		
I-II	7	2.2
III	44	14.0
IV	264	83.8
**Metastasis site**		
Brain	54	17.1
Bone	71	22.5
Liver	33	10.5
**Mutational status**		
EGFR	59	18.7
ALK	15	4.8
Negative	79	25.1
Unknown	162	51.4
**PD-L1 expression levels**		
<1%	109	34.6
1–49%	77	24.4
≥ 50%	67	21.3
Unknown	62	19.7
**Corticosteroid interference**		
Yes	13	4.1
No	302	95.9
**Abx treatment**		
Yes(≥7 days)	43	13.7
Yes(<7 days)	48	15.2
No	224	71.1
**Lines of therapy**		
1	165	52.4
2	150	47.6
**Immune drugs**		
atezolizumab	20	6.3
durvalumab	16	5.1
sintilimab	108	34.3
pembrolizumab	38	12.1
camrelizumab	46	14.6
tislelizumab	59	18.7
toripalimab	28	8.9

The median age of all patients was 63 years (range 27–85), including 254 males (80.6%) and 61 females (19.4%). A total of 216 patients were smokers (68.6%), and 285 patients (90.5%) had a ECOG PS < 2 at the time of diagnosis. By histology, 135 patients (42.9%) had adenocarcinoma, 117 (37.1%) had squamous cell carcinoma, and most patients (83.8%) had stage IV disease. Distant metastasis was identified in 158 patients (50.2%), and the most common site of metastasis was bone (22.5%), followed by brain (24.4%) and liver (10.5%). Of the patients undergoing genetic testing, only 74 patients (23.5%) carried genetic mutations. In terms of PD-L1 expression level, 67 cases (21.3%) were ≥ 50%, 77 cases (24.4%) were 1–49%, and 109 cases (34.6%) were less than 1%. Thirteen patients (4.1%) were treated with corticosteroids for more than 20 days, and 302 patients (95.9%) had no corticosteroid interference. Forty-three patients (13.7%) received Abx treatment for more than 7 days, and 48 patients (15.2%) received Abx for less than 7 days. A total of 165 patients (52.4%) received ICIs as the first-line application, while 150 patients (47.6%) received ICIs as the second-line application. A total of 315 patients received seven types of ICIs in the past; the most used was sintilimab in 108 cases (34.3%), and the least used was durvalumab in 16 cases (5.1%) (Table 1).

### Treatment plan and treatment cycle

All 315 patients received two or more cycles of ICIs. This study focused on patients with stage III-IV adenocarcinoma and Squamous cell carcinoma. One hundred thirty-two patients received with ICI as the first-line treatment, 25 received ICIs combined with chemotherapy plus anti-angiogenic therapy (A1), 82 received ICIs plus chemotherapy (A2), 11 received ICIs plus anti-angiogenic therapy (A3), 14 patients received ICIs alone (A4). The median treatment cycle of first-line treatment was eight cycles (2–20 cycles); 113 patients received ICI treatment as second-line treatment, 17 received ICIs combined with chemotherapy plus anti-angiogenic therapy (B1), 43 received ICIs plus chemotherapy (B2), 30 received ICIs plus anti-angiogenic therapy (B3), 23 patients received ICIs alone (B4). The median treatment cycle of second-line treatment was five cycles (2–10 cycles) (Table 2).

**Table 2:** Correlations between the clinical features of different treatment modalities with ORR and PFS

First-line(***n*** = 132)	n	CR	PR	SD	PD	ORR(%)	DCR(%)	Median PFS (months)	P
**Treatment pattern**									
A1	25	0	7	15	3	28.0	88.0	9.8	0.689
A2	82	0	34	45	3	41.5	96.3	11.4	
A3	11	0	4	6	1	36.4	90.9	10.2	
A4	14	0	6	7	1	42.9	92.9	16.1	
**Efficacy**									
2-cycle	132	0	51	73	8	38.6	93.9	11.40	
4-cycle	85	0	38	41	6	44.7	92.9		
**Histology**									
Adenocarcinoma	69	0	27	39	3	39.1	95.7	11.4	0.839
Squamous cell carcinoma	63	0	24	34	5	38.1	92.1	10	
**Mutational status**									
EGFR	28	0	10	16	2	35.7	92.9	12.0	0.767
ALK	5	0	2	3	0	40.0	100	not reached	
Negative	26	0	12	12	2	46.2	92.3	16.0	
Unknown	73	0	27	42	4	40.0	94.5	9.9	
**PD-L1 expression levels**									
<1%	38	0	11	21	6	28.9	84.2	6.0	0.000
1–49%	36	0	14	20	2	38.9	94.4	12	
≥ 50%	28	0	17	11	0	60.7	100	not reached	
Unknown	30	0	9	21	0	30.0	100	10.3	
**Corticosteroid interference**									
Yes	5	0	3	1	1	60.0	80.0	not reached	0.281
No	127	0	48	72	7	37.8	94.5	11.4	
**Abx treatment**									
Yes(≥7 days)	21	0	5	11	5	23.8	76.2	4	0.000
Yes(<7 days)	19	0	7	12	0	36.8	100	not reached	
No	92	0	39	50	3	42.4	96.7	13.6	
**Immune drugs in NSCLC**									
atezolizumab	6	0	3	3	0	50.0	100	9.0	0.188
durvalumab	3	0	2	1	0	66.7	100	not reached	
sintilimab	41	0	14	27	0	34.1	100	9.1	
pembrolizumab	18	0	9	8	1	50.0	94.4	16.0	
camrelizumab	17	0	5	8	4	29.4	76.5	8.3	
tislelizumab	33	0	14	17	2	42.4	93.9	10.0	
toripalimab	14	0	4	9	1	28.6	92.9	11.4	
**Second-line(*n*** **= 113)**									
**Treatment pattern**									
B1	17	0	6	10	1	35.3	94.1	8.0	0.283
B2	43	0	12	29	2	27.9	95.3	15.0	
B3	30	0	13	14	3	43.3	90.0	8.5	
B4	23	0	5	16	2	21.7	91.3	10.8	
**Efficacy**									
2-cycle	113	0	36	69	8	31.9	92.9	10.0	
4-cycle	71	0	13	50	8	18.3	88.7		
**Histology**									
Adenocarcinoma	64	0	19	38	7	29.7	89.1	10	0.117
Squamous cell carcinoma	49	0	17	31	1	34.7	98.0	18.1	
**Mutational status**									
EGFR	24	0	5	15	4	20.8	83.3	8.5	0.356
ALK	8	0	5	3	0	62.5	100	6.3	
Negative	43	0	11	29	3	25.6	93.0	12.7	
Unknown	38	0	15	22	1	39.5	97.4	10	
**PD-L1 expression levels**									
<1%	39	0	13	21	5	33.3	87.2	6.4	0.001
1–49%	26	0	3	22	1	11.5	96.2	13.4	
≥ 50%	27	0	12	14	1	44.4	96.3	not reached	
Unknown	21	0	8	12	1	31.0	96.6	6.4	
**Corticosteroid interference**									
Yes	3	0	2	1	0	66.7	100	4.5	0.025
No	110	0	34	68	8	30.9	92.7	10.8	
**Abx treatment**									
Yes(≥7 days)	14	0	4	8	2	28.6	85.7	6	0.002
Yes(<7 days)	18	0	6	12	0	33.3	100	not reached	
No	81	0	26	49	6	32.1	92.6	10.8	
**Immune drugs in NSCLC**									
atezolizumab	6	0	2	4	0	33.3	100	8.0	0.830
durvalumab	5	0	0	5	0	0	100	9.7	
sintilimab	45	0	14	29	2	31.1	95.6	13.4	
pembrolizumab	13	0	9	3	1	69.2	92.3	10.0	
camrelizumab	16	0	1	14	1	6.3	93.8	12.7	
tislelizumab	15	0	3	11	1	20.0	93.3	not reached	
toripalimab	13	0	7	3	3	53.8	76.9	8.5	

### Efficacy

**Fig. 1:**
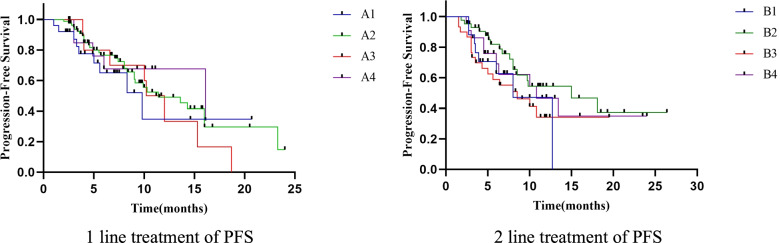
Kaplan-Meier survival curve of PFS

We mainly observed short-term efficacy (2 cycles of treatment). In patients with stage III-IV adenocarcinoma and Squamous cell carcinoma, 87 patients achieved PR, 142 patients presented SD, and the ORR and DCR of 245 patients were 35.5% (87/245) and 93.5% (229/245), respectively. The ORRs of ICI as the first-line and second-line treatment were 38.6% (51/132) and 31.9% (36/113), and the DCRs were 93.9% (124/132) and 92.9% (105/113), respectively (Table 2, Fig. 1).

### Analysis of the efficacy of first-line treatment

In the first-line treatment, the median PFS in Groups A1, A2, A3, and A4 was 9.8 m, 11.4 m, 10.2 m, and 16.1 m, respectively (*P* > 0.05). Compared adenocarcinoma with squamous cell carcinoma, the difference in median PFS was no statistically significant (*P* > 0.05). The median PFS, ORR, and DCR with ALK mutations were better than those with EGFR mutations, probably because the number of patients with ALK mutations was too small. Among patients with EGFR mutations, there were 9 in group A1, 15 in group A2, 2 in group A3, 2 in group A4. The median PFS was 8.3 m, 13.6 m, 13.7 m, and 10.5 m, respectively (*P* = 0.066). In terms of PD-L1 expression, high PD-L1 expression compared with low PD-L1 expression prolonged the median PFS, and the differences in median PFS were statistically significant (*P* < 0.05). During the first-line application of ICIs treatment, no significant difference was found in median PFS between patients with and without corticosteroid interference(*P* > 0.05), while we observed a statistically significant improvement in the median PFS of 4 months in patients with Abx treatment> 7 days, not reached in patients with Abx treatment < 7 days, and 13.6 months in patients without Abx treatment (*P* < 0.05).

### Analysis of the curative effect of second-line treatment

In the second-line treatment, the median PFS in Groups B1, B2, B3, and B4 was 8.0 m, 15.0 m, 8.5 m, and 10.8 m, respectively (*P* > 0.05). There were no differences in median PFS according to pathological type and gene mutation type (*P* > 0.05). Among patients with EGFR mutations, there were 4 in group B1, 7 in group B2, 6 in group B3, 7 in group B4. The median PFS was 3.4 m, 8.5 m, 2.5 m, and 10.8 m, respectively (*P* = 0.647). In terms of PD-L1 expression, the median PFS of <1%, 1–49%, ≥50% and undetected group was 6.4 months, 13.4 months, not reached and 6.4 months, respectively. The differences were statistically significant (*P* < 0.05). In terms of the effects of corticosteroids and Abx, we also found significant differences in median PFS (*P* < 0.05).

### Subgroup analysis

The results of subgroup analysis were mainly to analyze the short-term efficacy in patients with stage III-IV adenocarcinoma and Squamous cell carcinoma, and the median PFS also provided evidence for subgroup differences. ECOG PS, EGFR mutations, pathological type and number of treatment lines were not correlated with the median PFS (*P* > 0.05). In terms of PD-L1 expression, corticosteroid interference and Abx treatment, the differences between the groups were statistically significant (*P* < 0.05). PD-L1 expression was ≥50% compared with 1–49 and < 1%, and the median PFS was longer (not reaching vs. 13.4 months vs. 6.4 months). No corticosteroid interference was longer than with corticosteroids (≥20 days) in median PFS (11.4 months vs. 9.0 months), and the group without Abx treatment had a longer median PFS compared with Abx treatment for < 7 days and ≥ 7 days (13.4 months vs. 12.7 months vs. 5.0 months) (Table 3).

**Table 3:** Univariate analysis of the correlation between clinical features and PFS

Variable	N	Median PFS (months)	P
**ECOG PS**			
0–1	220	11.7	0.159
2	25	10.2	
**EGFR** mutations			
Yes	52	10.8	0.212
No	69	15.0	
**PD-L1 expression levels**			
<1%	77	6.4	0.000
1–49%	62	13.4	
≥ 50%	55	not reached	
Unknown	59	9.1	
**Histology**			
Adenocarcinoma	133	10.8	0.086
Squamous cell carcinoma	112	14.3	
**Corticosteroid interference**			
Yes	8	9.0	0.015
No	237	11.4	
**Abx treatment**			
Yes(≥7 days)	35	5.0	0.002
Yes(<7 days)	37	12.7	
No	173	13.4	
**Lines of therapy**			
1	132	11.4	0.707
2	113	10.0	

### Adverse events

The overall incidence of adverse events in 315 patients was 62.5% (197/315), the incidence of irAEs was 13.7% (43/315), and grade 1–2 and grade 3–4 adverse events were 34.9 and 27.6%, respectively. The most common adverse reactions were leukopenia (54.9%), anemia (41.6%), and fatigue (32.4%), which did not have much effect on the progress of treatment. There were four patients with grade 5 irAEs that eventually resulted in death: two cases had liver damage leading to liver failure, one case had immune-related pneumonia, and one case had immune-related myocarditis (Table 4).

**Table 4:** Treatment-related adverse events

Adverse events	Grade I-II	Grade III-IV	Grade V	Percentage
**Fatigue**	102	0	0	32.4
**Nausea**	25	2	0	8.6
**Diarrhoea**	37	1	0	12.1
**Skin rash**	10	0	0	3.2
**Glu Glutamic oxaloacetic transaminase/Alanine aminotransferase elevation**	15	3	2	5.7
**Renal dysfunction**	8	0	0	2.5
**Leucopenia**	93	80	0	54.9
**Anaemia**	86	45	0	41.6
**Thrombocytopenia**	31	18	0	15.6
**Pneumonitis**	25	10	1	11.1
**Myocarditis**	8	2	1	3.5
**Endocrine toxicity**	3	0	0	1

### Prognosis

As of the last follow-up time on March 31, 2021, a total of 15 patients were lost to follow-up, with a loss rate of 4.8%. The median PFS was 10.8 months, and the median OS was not reached. Fifty-six patients died, of which 52 patients died of tumor progression, and four patients died of severe irAEs. The one-year survival rate was 93.3%. By the end of the last follow-up time, 80 patients had discontinued ICIs due to disease progression or side effects, while 164 patients were still receiving ICIs with stable conditions.

## Discussion

At present, immunotherapy is regarded as a new revolution in the treatment of malignant tumors, opening up a new therapeutic field for patients with advanced lung cancer. The programmed death cell protein 1 (PD-1) inhibitors nivolumab and pembrolizumab, as well as the PD-L1 inhibitors atezolizumab and durvalumab, have been widely used in clinical practice. Nivolizumab, pembrolizumab, and atezolizumab have been approved by the FDA for advanced NSCLC [9]. In recent years, a number of clinical trials have confirmed that compared with chemotherapy, ICIs has a significant survival advantage and has become the new standard treatment for advanced lung cancer. Arrieta O et al. published in JAMA Oncology the first prospective randomized clinical trial of pembrolizumab combined with docetaxel in the second-line treatment of advanced NSCLC patients who progressed after chemotherapy. The results show that immunotherapy combined with chemotherapy significantly improved the ORR and PFS compared to chemotherapy alone [10]. In the KEYNOTE-024 clinical study, pembrolizumab was used to treat patients with advanced lung cancer. Its PFS was 10.3 months, which was significantly longer than that of chemotherapy, and the risk of death was also reduced by 40% [11]. The KEYNOTE-010 [6] trial randomly assigned patients with PD-L1 expression ≥1% to be treated with different doses of pembrolizumab compared with docetaxel chemotherapy. Patients treated with 2 mg/kg and 10 mg/kg pembrolizumab at different doses had better OS (10.4 months vs. 12.7 months vs. 8.5 months) than those treated with chemotherapy. In 2018, the results of the IMpower131 study showed that regardless of PD-L1 expression, ateliizumab combined with chemotherapy as a first-line treatment can prolong the PFS of patients [12]. IMpower150 [8] is a multicenter, open clinical study, which is the first phase III clinical trial that combines ICIs with antiangiogenic therapy and chemotherapy for the first-line treatment of advanced nonsquamous NSCLC, including 1202 patients. The results showed that atelizumab combined with antiangiogenic therapy and chemotherapy provided significant PFS benefits in advanced NSCLC patients with EGFR or ALK mutations, low Teff expression, PD-L1 negativity, and liver metastasis compared with the control group (anti-angiogenesis combined chemotherapy), providing evidence of the effectiveness of the combination therapy, and the safety risks were consistent with previously reported single-agent therapy. Numerous clinical studies have shown that ICIs has a good therapeutic effect on NSCLC patients in the real world. In this study, the median PFS of the patients who took ICIs as the first-line or second-line treatment was significantly longer than that in previous clinical trials.

Among the patients collected in our study, there were fewer EGFR mutation-positive patients, and the median PFS showed no significant difference between the EGFR mutations and the subgroup without mutations. Checkmate057 [4] and KEYNOTE-010 [6], these two clinical trial subgroup analysis results showed the comparison of ICIs compared with chemotherapy for advanced NSCLC in the EGFR mutation group, there was no significant difference in OS, and EGFR mutation patients did not benefit from immunotherapy and were not sensitive to immunotherapy. The possible mechanism is that EGFR mutation is related to inhibiting the tumor microenvironment and reducing the tumor mutation burden [13]. In the subgroup analysis of the IMpower150 trial, 80 patients with EGFR-positive mutations who had previously failed EGFR-TKI treatment had a median PFS of 10.2 months after receiving immunotherapy combined with chemotherapy and antiangiogenesis therapy. This suggests that ICI combined with chemotherapy and antiangiogenic therapy may benefit patients with advanced NSCLC who have EGFR mutations [8]. Whether advanced lung cancer patients with EGFR mutations can benefit from immunotherapy has not been concluded at present, and more targeted clinical trial data are still needed.

In the treatment of lung cancer patients, they often face the use of corticosteroids, such as treatment of brain metastases to reduce and prevent brain edema and improve tumor-related complications, the treatment of moderate to severe irAEs, and pretreatment before ICIs combined with chemotherapy. Some relevant clinical studies have confirmed that the application of corticosteroids during ICI treatment may inhibit the antitumor efficacy of ICIs [14–16]. The retrospective study conducted by Kathryn et al. [17] included 640 patients treated with ICIs monotherapy in two cancer centers, of which 90 patients received corticosteroid therapy (≥10 mg/d prednisone). The application of corticosteroids affects the efficacy benefit of ICIs and significantly reduces the PFS and OS of NSCLC patients. In the Memorial Sloan Kettering Cancer Center (MSKCC) cohort, corticosteroid use was associated with a shorter median PFS (1.9 months vs 2.6 months) and a shorter median OS (5.4 months vs 12.1 months). In the Gustave Roussy Cancer Center (GRCC) cohort, the median PFS (1.7 months vs 1.8 months) and median OS (3.3 months vs 9.4 months) were also significantly reduced in those receiving corticosteroid therapy. The results of this study showed that the use of corticosteroids shortened the median PFS, which was consistent with the results of previous retrospective studies.

Abx further interferes with the development of the system’s immune response by affecting the function of T cells, changing the production of cytokines, and interfering with the role of dendritic cells (DCs) [18, 19]. In the study of immunotherapy, Abx is mainly used for infection of the lung, urinary system, skin, and other parts. Caicun Zhou et al. [20] reported a negative correlation of Abx treatment with PFS and OS. The study of Hakozaki et al. [21] showed that NSCLC patients who received Abx before nivolumab treatment had a shorter median PFS and median OS than patients without Abx use (median PFS: 1.2 months vs. 4.4 months, median OS: 8.8 months vs. not reached), and the differences were statistically significant (all *P* < 0.05), but multivariate analysis showed that Abx treatment was not an independent predictor of PFS. A large retrospective study reported that patients who received multiple courses or continuous use of Abx for more than 7 days had a shorter median OS and significantly reduced PFS [22]. The study emphasizes that patients with multiple or long-term use of Abx have worse ICIs treatment effects and aims to advocate that we use Abx with caution in the clinic. Our research also confirmed that Abx will affect the efficacy of ICIs regardless of whether ICIs are first-line or second-line treatment, and it was speculated that the length of Abx treatment was negatively correlated with the length of the median PFS. However, the effect of Abx treatment on the efficacy of ICIs remains to be further evaluated.

PD-L1 expression is a marker related to the efficacy of first-line immunotherapy recommended by the NCCN guidelines. KEYNOTE-024 [3] is a comparative study of the efficacy of pembrolizumab treatment and standard platinum-containing chemotherapy for initially treated advanced NSCLC. The study included patients with PD-L1 expression ≥50%. Compared with the chemotherapy group, the ICIs group had a higher ORR, longer PFS (10.3 months vs 6.0 months), longer OS (30.0 months vs 14.2 months), and a lower incidence of adverse reactions (73.4% vs 90%). However, IMpower132 [23] and KEYNOTE-189 [7] compared the efficacy of ICIs combined with chemotherapy versus chemotherapy in patients with advanced NSCLC, and the results showed that ICIs improved PFS and OS regardless of PD-L1 expression. In the study using ICIs as second-line treatment, KEYNOTE-010 [6] was the first prospective study to demonstrate that PD-L1 expression is an important biomarker for predicting the efficacy of ICIs. These results indicated that the OS and PFS of patients with high PD-L1 expression were prolonged compared with those with low PD-L1 expression in second-line treatment. However, for patients with advanced NSCLC included in CheckMate 017 [24] and OAK [25] who received second-line immunotherapy compared with chemotherapy, the level of PD-L1 expression could not predict the efficacy. Compared with the second-line population, the median PFS of the population who used ICIs as the first-line treatment in this study was prolonged. The analysis of this study showed that the median PFS with PD-L1 expression ≥50% was better than 1–49 and < 1% of patients in the treatment group, and there was a significant difference. Both this study and KEYNOTE-010 suggest that patients with high PD-L1 expression may be more likely to benefit from ICI treatment.

With the development of an increasing number of clinical trials, the adverse reactions of chemotherapy and immunotherapy have received increasing attention from researchers. ICIs act on immune cells, causing some normal cells of the body to be attacked to produce irAEs, which is essentially an inflammatory reaction caused by excessive activation of the immune system [26]. Common adverse reactions caused by chemotherapy mainly include fatigue, nausea, vomiting, and bone marrow suppression. IrAEs can affect multiple systems, the most common of which are the skin, lungs, gastrointestinal tract, and endocrine system, and relatively rare occurrence sites are the nervous system and cardiovascular system [27, 28]. Most irAEs have mild symptoms, but myocarditis, pneumonia, hepatitis, and neurotoxicity may be fatal, requiring clinical vigilance and attention [29, 30]. In this study, the overall incidence of adverse events was 62.5% (197/315), the incidence of irAEs was 13.7% (43/315). There were four patients with grade 5 irAEs that eventually resulted in death: one case had immune-related pneumonia, one case had immune-related myocarditis, and two cases had liver damage leading to liver failure. One patient died of the infection associated with immunosuppression. The patient received 10 cycles of sintilimab. At the end of the treatment, he developed cough, chest tightness, and repeated fever. Re-examination of chest Computed Tomography (CT) revealed pneumonia, combined with multiple serous effusions and gastrointestinal hemorrhage, although he was given ventilator support and anti-infection, corticosteroid and symptomatic treatment and eventually died of severe lung infection and respiratory failure. Immune-related myocarditis is a rare but relatively fatal irAE that manifests as increased troponin, creatine kinase, and brain natriuretic peptide (BNP) [31]. The incidence in this study was 3.5%, one patient developed myocarditis after two cycles of treatment with camrelizumab, and troponin continued to increase. After stopping the drug, corticosteroid therapy was still ineffective. Finally, the patient died of extensive myocardial injury. Myocarditis often has an early onset but has a high risk of death and should be identified and treated as soon as possible [32]. The incidence of irAEs is usually low, but once they cause adverse reactions of important organs, they may seriously endanger the lives of patients. The research data for irAEs remain incomplete, and there is a lack of effective methods to predict and screen patients with severe irAEs. In clinical work, we should detect and deal with irAEs as early as possible, cooperate with multiple disciplines, minimize risks, and strive to ensure the best benefit for patients. Two patients who received sintilimab and camrelizumab developed liver damage, continued increases in transaminase, combined with ascites, and later developed hepatic encephalopathy and eventually died due to disease progression, liver failure, and respiratory failure.

## Conclusions

In the real world, ICIs has a good effect on patients with advanced lung cancer and significantly improves ORR and PFS. How to choose the best combination therapy drug plan, how to screen the patient groups that may benefit the most, and how to choose evaluation indicators that are effective for prognosis all require future research and trials.

## Data Availability

The datasets generated and/or analysed during the current study are not publicly available due these data will be prepared for further studies, we will release relevant data when all the studies are completed, but are available from the corresponding author on reasonable request. To request data please contact Prof. Yu Li (email: qlliyures@163.com).
